# Genomic profiling of breast tumours in relation to *BRCA *abnormalities and phenotypes

**DOI:** 10.1186/bcr2334

**Published:** 2009-07-09

**Authors:** Olafur Andri Stefansson, Jon Gunnlaugur Jonasson, Oskar Thor Johannsson, Kristrun Olafsdottir, Margret Steinarsdottir, Sigridur Valgeirsdottir, Jorunn Erla Eyfjord

**Affiliations:** 1 Faculty of Medicine, University of Iceland, Vatnsmyrarvegur 16, Reykjavik, Iceland; 2 Department of Pathology, Landspitali University Hospital, Hringbraut, Reykjavik, 101, Iceland; 3 Department of Oncology, Landspitali University Hospital, Hringbraut, Reykjavik, 101, Iceland; 4 Department of Genetics and Molecular Medicine, Landspitali University Hospital, Hringbraut, Reykjavik, 101, Iceland; 5 Roche NimbleGen, Inc., Vínlandsleið 2-4, Reykjavik, 113, Iceland

## Abstract

**Introduction:**

Germline mutations in the *BRCA1 *and *BRCA2 *genes account for a considerable fraction of familial predisposition to breast cancer. Somatic mutations in *BRCA1 *and *BRCA2 *have not been found and the involvement of these genes in sporadic tumour development therefore remains unclear.

**Methods:**

The study group consisted of 67 primary breast tumours with and without *BRCA1 *or *BRCA2 *abnormalities. Genomic alterations were profiled by high-resolution (~7 kbp) comparative genome hybridisation (CGH) microarrays. Tumour phenotypes were analysed by immunohistochemistry on tissue microarrays using selected biomarkers (ER, PR, HER-2, EGFR, CK5/6, CK8, CK18).

**Results:**

Classification of genomic profiles through cluster analysis revealed four subgroups, three of which displayed high genomic instability indices (GII). Two of these GII-high subgroups were enriched with either *BRCA1*- or *BRCA2*-related tumours whereas the third was not *BRCA*-related. The *BRCA1*-related subgroup mostly displayed non-luminal phenotypes, of which basal-like were most prominent, whereas the other two genomic instability subgroups *BRCA2*- and GII-high-III (non-*BRCA*), were almost entirely of luminal phenotype. Analysis of genome architecture patterns revealed similarities between the *BRCA1*- and *BRCA2 *subgroups, with long deletions being prominent. This contrasts with the third instability subgroup, not *BRCA*-related, where small gains were more prominent.

**Conclusions:**

The results suggest that *BRCA1*- and *BRCA2*-related tumours develop largely through distinct genetic pathways in terms of the regions altered while also displaying distinct phenotypes. Importantly, we show that the development of a subset of sporadic tumours is similar to that of either familial *BRCA1*- or *BRCA2 *tumours. Despite their differences, we observed clear similarities between the *BRCA1*- and *BRCA2*-related subgroups reflected in the type of genomic alterations acquired with deletions of long DNA segments being prominent. This suggests similarities in the mechanisms promoting genomic instability for *BRCA1*- and *BRCA2*-associated tumours, possibly relating to deficiency in DNA repair through homologous recombination. Indeed, this feature characterized both familial and sporadic tumours displaying *BRCA1*- or *BRCA2*-like spectrums of genomic alterations. The importance of these findings lies in the potential benefit from targeted therapy, through the use of agents leading to DNA double-strand breaks such as PARP inhibitors (olaparib) and cisplatin, for a much larger group of patients than the few *BRCA1 *and *BRCA2 *germline mutation carriers.

## Introduction

Germline mutations in the *BRCA1 *or *BRCA2 *genes significantly increase the risk of developing early-onset breast cancer [1]. Tumours derived from *BRCA1 *or *BRCA2 *germline mutation carriers have generally lost the wild-type *BRCA1 *or *BRCA2 *alleles, respectively [2,3]. These observations suggest important roles for the *BRCA1 *and *BRCA2 *genes as tumour suppressors. The *BRCA1 *and *BRCA2 *gene products are both phosphorylated by ATR (ataxia telangiectasia and Rad3 related) which, in turn, is activated by DNA damage and stalled replication forks [4,5]. BRCA1 is important in recruitment of various DNA repair proteins, including BRCA2, to sites of DNA damage, whereas BRCA2 is crucial for catalysing the formation of RAD51 filaments on single-stranded DNA at the damaged sites [6,7]. The BRCA1 and BRCA2 proteins are linked within a network of protein interactions having a common objective of responding to DNA damage and stalled replication forks [8]. Disruption of key elements within this network might explain why cells defective in either BRCA1 or BRCA2 display genomic instability and are sensitive to DNA damage that involves double-strand breaks [4]. This has suggested potential therapeutic applications through the use of agents that lead to double-stranded DNA breaks such as PARP inhibitors, mitomycin C and platinum salts [9].

The involvement of the *BRCA1 *and *BRCA2 *genes in sporadic breast tumour development has been questioned because somatic mutations in *BRCA1 *or *BRCA2 *have not been found [10,11]. However, methylation of the *BRCA1 *gene promoter and loss of *BRCA1 *gene expression are significantly associated and occur frequently in sporadic breast tumours [12]. We have previously reported on epigenetic silencing of the *BRCA1 *gene through promoter methylation in about 10% of an unselected set of sporadic breast cancers [13]. These observations suggest that epigenetic silencing of the *BRCA1 *gene might be an alternative to somatic mutations as a mechanism of *BRCA1 *inactivation in sporadic cases of breast cancer. In addition, it has been demonstrated that the *BRCA2 *gene is negatively regulated by protein interactions with gene products of the *EMSY *gene which, in turn, is frequently amplified in sporadic breast tumours [14]. This suggests an important link between the *BRCA2 *gene and sporadic tumour development.

Analyses of genomic and gene expression profiles in unselected sets of breast tumours have revealed subgroups of biological and clinical relevance [15,16]. These studies have shown that the expression profiles of tumours derived from *BRCA1 *germline mutation carriers strongly resemble those of sporadic basal-like tumours [17]. This has suggested that underlying *BRCA1 *abnormalities could promote sporadic basal-like tumour development. Supporting this notion is the finding that sporadic basal-like tumours frequently display a significantly reduced expression of the *BRCA1 *gene and genomic instability [18,19]. However, a subset of sporadic basal-like tumours do not display large-scale genomic instability which have been proposed to represent a novel subtype of breast cancers [20]. Here, we have profiled and examined the patterns of genomic alterations in familial *BRCA1 *and *BRCA2 *tumours in the context of sporadic tumours with and without epigenetic silencing of the *BRCA1 *gene. The results were coupled with analysis of tumour phenotypes using a selected set of biomarkers on tissue microarrays. We then specifically addressed the question of whether the *BRCA1 *and *BRCA2 *genes are involved in sporadic breast tumour development.

## Materials and methods

### Study group

The study group was derived from a well-defined population with respect to the local *BRCA1 5193G-> A *and *BRCA2 999del5 *germline mutations [21,22]. All patients within the study group had previously been screened for these *BRCA1 *and *BRCA2 *germline mutations. Sporadic tumours were defined as those derived from patients that were negative for the local *BRCA *germline mutations with no known family history of the disease.

The study group consisted of a selected set of primary infiltrating female breast tumour samples (n = 67). The samples were selected from tumours derived from *BRCA1 *and *BRCA2 *germline mutation carriers along with sporadic tumours with and without epigenetic silencing of the *BRCA1 *gene. At least one sporadic tumour (n = 38) without *BRCA *abnormalities was selected for each of the *BRCA *abnormal tumours (n = 29), that is, familial *BRCA1 *(n = 3) and *BRCA2 *(n = 18) tumours along with sporadic tumours displaying epigenetic silencing of the *BRCA1 *gene (n = 8). All tumours, sporadic and familial BRCA1 and BRCA2, were selected by their patient age at diagnosis of 61 years or younger. The DNA samples had previously been isolated from freshly frozen tumour tissue and these samples were obtained from the Biological Specimen Bank of the Icelandic Cancer Society. The tumour samples were macroscopically examined prior to DNA isolation and portions showing viable tumour tissue were identified. These portions were then selected for DNA isolation, which was performed using a standard phenol-chloroform plus proteinase K protocol. Data on clinical parameters were obtained from the Department of Pathology and Department of Oncology, Landspitali Hospital, Reykjavik, Iceland. Time to relapse refers to the time from surgical removal of the primary tumour to diagnosis of recurrence or metastasis. This work was carried out according to permits from the Icelandic Data Protection Commission (2006050307) and Bioethics Committee (VSNb2006050001/03-16). Informed consent was obtained from all patients.

### Array comparative genomic hybridisation

Comparative genomic hybridisation (CGH) was performed using high-resolution oligonuclueotide microarrays (Roche NimbleGen, Inc., Reykjavik, Iceland) [23]. The arrays used, "2006_11_01 HG17_WG_CGH" (n = 46) and "080101 HG18_WG_CGH_v2_X1" (n = 21), were of a standard design developed by Roche NimbleGen, Inc. (Reykjavik, Iceland) covering the human genome in about 7 kbp median resolution. Sample preparations and hybridisations were carried out according to manufacturer's protocols (NimbleGen Arrays User's Guide-CGH Analysis, Roche NimbleGen, Inc., Reykjavik, Iceland). Cy3 and Cy5 signal intensity distributions were then normalized using the qspline method (affy package in Bioconductor for R) [24]. The array CGH data are available in the ArrayExpress repository (E-TABM-712).

### Methylation specific PCR and allelic imbalance

Methylation at the *BRCA1 *promoter region was assessed in all tumours within the study group by methylation-specific PCR (MSP) as previously described [13]. Allelic imbalance by microsatellite analysis at the *BRCA1 *and *BRCA2 *loci had previously been performed [13].

### Tissue microarrays and expression analysis

Core samples were removed (1.0 mm diameter) from each tumour and rearranged on empty paraffin-blocks using a manual tissue microarray device (BEECHER MTA II; Beecher Instruments, Inc., Sun Prairie, Wisconsin, USA).

Immunohistochemistry (IHC) was applied to 4 μm thick tissue microarray (TMA) sections mounted on superfrosted slides (Menzel, Germany). The slides were dewaxed and immerged in Tris-EDTA, pH 9, (HIER) in microwave oven at 99°C. Endogenous peroxidase activity was inactivated by incubation in blocking solution and the slides then incubated with primary antibody (30 minutes). Polymer conjugate was used as Visualization System (K4061, EnVision+ Dual Link, DAKO, DK-2600, Glostrup, Denmark) (30 minutes) and DAB (K-3468, DAKO, DK-2600, Glostrup, Denmark) used as chromogen (10 minutes).

Expression analysis by IHC on TMA sections was performed for oestrogen receptor (ER), progesterone receptor (PR), human epidermal growth factor receptor (HER)-2, epidermal growth factor receptor (EGFR), cytokeratin (CK) 5/6, CK8, CK18 and BRCA1 [see Additional data file 1]. Expression levels were estimated blindfolded to previously established tumour characteristics and *BRCA *abnormalities. Expression of ER and PR were scored on a discontinuous scale of 0+, 1+, 2+ and 3+ with the addition of hyper-intense staining (> 3+) being remarked for those tumours displaying extremely intense and concentrated nuclear staining. Positive nuclear ER and PR immunostaining was defined as any visible staining in more than 1% of tumour cell nuclei. Information obtained from Landspitali Hospital, Department of Pathology (ligand binding assay) was used to complement missing data on ER and PR expression thereby allowing ER and PR positivity/negativity to be established for all tumours within the study group. HER-2 positivity was defined as staining of tumour cellular membranes displaying a score of 3+ according to criteria provided by the manufacturer (DAKO, DK-2600, Glostrup, Denmark). EGFR was scored on a discontinuous scale of 0+, 1+, 2+ and 3+ which was estimated by the staining intensity of tumour cellular membranes following descriptions provided by the manufacturer (DAKO, Glostrup, Denmark). EGFR positivity was defined as tumours displaying any, weak or strong, staining of the cellular membrane whereas a score of 2+ or higher was defined as high EGFR expression. Expression of BRCA1 was estimated by nuclear staining where loss of BRCA1 expression was defined as no visible nuclear staining whereas positive expression was defined as any visible, weak or strong, nuclear staining. The CK5/6 marker was scored as positive when weak or strong cytoplasmic and/or membranous staining was visible and otherwise scored as negative. CK8 and CK18 were scored on a scale of 0+, 1+, 2+ and 3+ according to descriptions provided by the manufacturer (DAKO, Glostrup, Denmark).

### Definition of tumour phenotypes and BRCA abnormalities

Luminal phenotype was defined as positivity for either ER or PR whereas non-luminal phenotype was defined as negativity for both ER and PR. The five biomarker classification scheme given in Cheang and colleagues was used to further subdivide these two phenotypic categories into luminal, luminal-HER2, 5NP (five-marker negative phenotype), non-luminal HER2 and basal-like phenotypes [25].

Tumours derived from *BRCA1 *and *BRCA2 *germline mutation carriers were defined as *BRCA1 *and *BRCA2 *abnormal, respectively. Additionally, tumours displaying epigenetic silencing of the *BRCA1 *gene were defined as *BRCA1 *abnormal in those cases where *BRCA1 *promoter methylation was coupled with complete absence of nuclear BRCA1 protein expression.

### Fluorescence *in situ *hybridisation

Fluorescence *in situ *hybridisation (FISH) was performed on paraffin-embedded and formalin-fixed tumour tissue sections (4 μm). DNA probe specific for the EMSY gene (BAC human CTD 2501F13, Invitrogen, Carlsbad, CA, USA) was labelled with SpectrumOrange-dUTP (Vysis, Des Plaines, IL, USA) by nick translation, and pRB11 clone for the centromere of chromosome 11 labelled with fluorescein-12-dUTP (Enzo Life Sciences, Farmingdale, NY, USA). Slides were deparaffinised and pretreated, probes and cotI DNA denatured in t-DenHyb-2 hybridisation buffer (Insitus Biotechnologies, Albuquerque, NM, USA) and hybridised to the tumour sections overnight. Stringency wash was performed at 72°C in solution containing 2 × SSC/0.3% NP-40. Analysis was performed in a Leica DMRXA2 fluorescence microscope with at least 100 cell nuclei counted in each experiment. Thresholds for copy gains were set at gene/centromere ratio of more than 1.5 and high-level amplification at a ratio of more than 2.5.

### Statistical analysis and data mining of array-CGH data

The Cy3/Cy5 ratio signal intensities were log2 transformed following normalisation of each array CGH experiment. The data were then represented by the median of log2 ratios within a window of five probes resulting in a median resolution of about 37.5 kb. Copy number alterations were identified by the Circular Binary Segmentation (CBS) algorithm implemented in DNAcopy (Bioconductor for R) with an alpha of 0.01 to identify change points while cancelling splits having less than 1.0 standard deviation units in difference through the sd.undo procedure [24,26]. The threshold for determining copy number alterations was fixed at ± 0.08, which was selected to capture the level of plateaus above and below the baseline as observed within the examined tumour genomes [26]. To further refine these thresholds we obtained estimates of probe noise levels for each of the arrays as described in Fridlyand and colleagues [27] to subsequently categorise the arrays by their noise levels with the discriminators being the lower and upper quartiles of the distribution. The thresholds for arrays displaying high and low noise levels were then modified to ± 0.10 and ± 0.06, respectively. The assigned thresholds were then validated by examining the association between immunostaining scores of the *HER-2 *gene (HercepTest, DAKO, DK-2600, Glostrup, Denmark) and copy number states, that is, gains, no change and deletions, which was found to be highly significant (Pearson's correlation, r = 0.53, *P *= 3.2 × 10^-5^). Additionally, we found the frequency of copy number gains at the *HER-2 *gene locus to be 27% (18 of 67), which is in line with previous reports [20]. High-level amplifications were defined as segment means that exceed 2SD units above the mean of log_2 _ratios derived from segments gained in copy number. These thresholds were validated by examining the association between HER-2 overexpression (HercepTest, Score 3+) and high-level amplification of the *HER-2 *gene (Pearson's correlation, r = 0.64, *P *= 1.3 × 10^-7^).

The CBS output was then used to represent each of the tumour genomes as segmented profiles in terms of copy number states, that is, +1 for copy gains, 0 for no change and -1 for deletions. Copy number alterations often cover large genomic regions within which a subset of one or more genes may be targeted [28]. Thus, classification of genomic profiles is subject to a large degree of inherent biological noise. This was addressed by projecting the data to lower dimensions through principal component analysis (PCA) prior to cluster analysis making use of only the first few components. This was performed in R 2.7.2 where the prcomp function was used to obtain the components. The first three principal components explained about 40% of the variability in the genomic data [see Additional data file 2]. Each of the other components explained less than 5% of the variability and were ignored in the subsequent steps. Cluster analysis was performed on the lower-dimensional data using the *k*-means cluster algorithm in R 2.7.2. This was carried out in an iterative procedure where the silhouette information was estimated using the silhouette function in R 2.7.2 (cluster package) to establish strong and reproducible results. Average silhouette information was used to identify the number of clusters in the data with 1000 iterations for each *k *= {2, 3, ..., 10}. Tumours with a low silhouette information (silhouette < 0.20) were considered borderline instances and were classified by first filtering the data in a Kruskal-Wallis hypothesis test (*P *< 0.01) while leaving out all such borderline tumours and then determining their membership position by re-applying the cluster procedure. A final model was then derived through the same procedure resulting in all tumours being assigned a cluster membership indicator.

To compensate for the few samples derived from *BRCA1 *germline mutation carriers in our study we obtained previously published array CGH data available online through ArrayExpress (E-TABM-170). This dataset included genomic profiles derived from five familial *BRCA1 *tumours, which were combined with our dataset. These five familial *BRCA1 *tumours were analysed by first identifying copy number alterations as described in Fridlyand and colleagues [27]. The output was then used to represent each of the tumour genomes as segmented profiles in terms of copy number states as described above. These segmentation profiles were then combined with our dataset by obtaining copy number states from each of the tumour genomes analysed in this study representing the nearest genomic region to those represented on the CGH arrays used in the Fridlyand and colleagues study [27]. This was performed by determining the difference in genomic length for each location between the two array platforms and then selecting the minimal distance. This procedure reduces the median array resolution from about 7 kb to about 765 kb, that is, from the NimbleGen high-resolution design to that used in Fridlyand and colleagues [27].

The degree of genomic instability for each tumour was estimated by determining the fraction of the genome altered. This was computed by obtaining copy number states for each of the windowed probes and determining the number of those assigned as altered in copy number against the total number of windowed probes. This measure, referred to as the genomic instability index (GII), has been described previously [20].

Genomic alterations characterising each of the subgroups were identified by using a conservative modification of the Fisher's exact test with *P *≤ 0.001, which was applied on the filtered dataset. This conservative modification of the Fisher's exact test has the advantage of penalising low *P*-values based on few counts [29].

## Results

### Copy number alterations in breast tumour genomes

**Figure 1:**
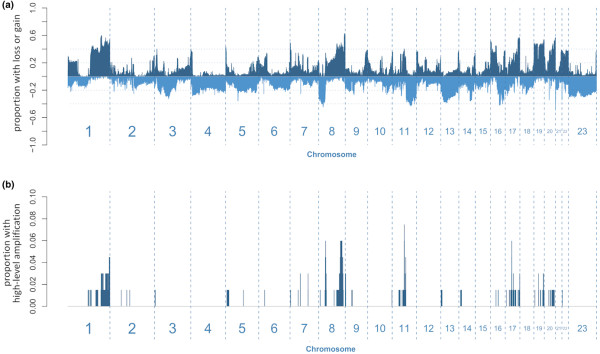
Genomic alterations within the study group. **(a) **The proportion of tumours with copy gains (positive) or deletions (negative) plotted along each chromosome. **(b) **The proportion of tumours with high-level amplifications plotted along each chromosome.

Genomic alterations present within the study group were visualised by generating a frequency plot displaying the proportion of tumours with copy number gains and deletions at each genomic location analysed (Figure 1a). Examination of the frequency plot reveals that regions frequently gained are infrequently deleted and *vice versa*. It can also be seen that sites of recurrent high-level amplification events occur within genomic regions that are frequently gained in copy numbers (Figure 1b). These observations show that copy number alterations are not randomly distributed throughout the tumour genomes.

### Classification of genomic profiles

Variability present in the spectrum of genomic alterations within the study group was examined by unsupervised classification of the genomic profiles through cluster analysis. The purpose was to examine the resulting tumour subgroups in terms of their prevalence for *BRCA1 *and *BRCA2 *abnormalities.

**Figure 2:**
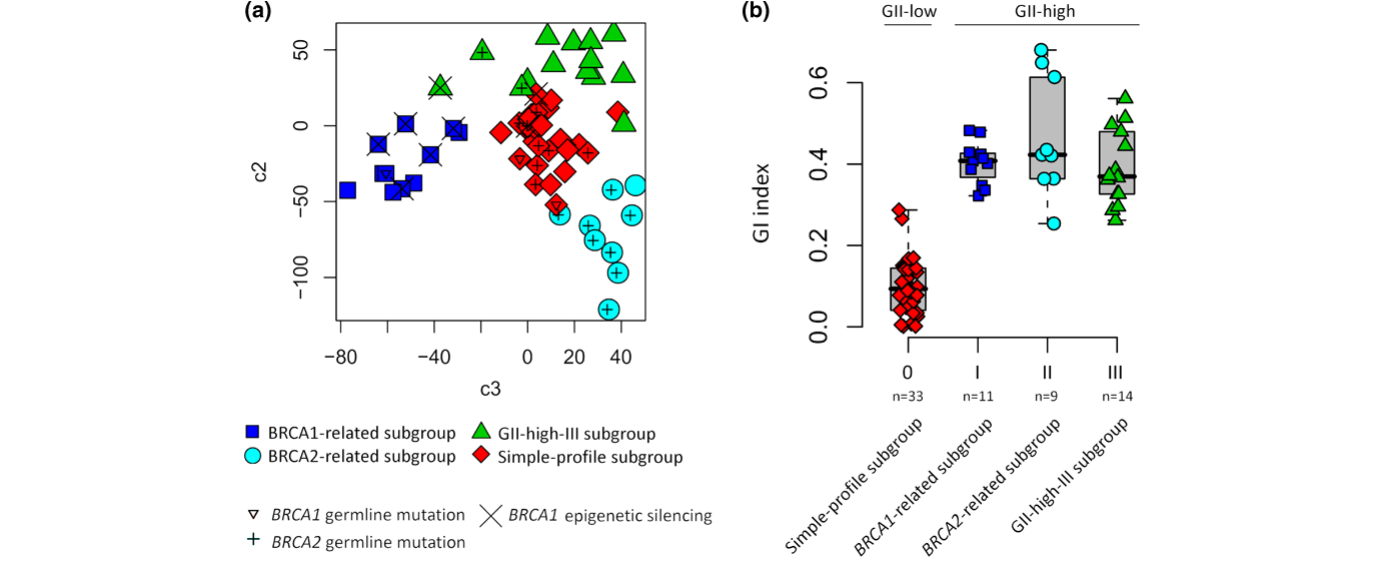
Classification of breast tumours by their genomic profiles through cluster analysis identified four distinct subgroups. **(a) **Cluster membership outcomes are visualised in terms of *BRCA *abnormalities through principal component analysis (PCA). Two of the four identified subgroups were enriched for either *BRCA1 *or *BRCA2 *abnormalities referred to as the *BRCA1*-related (n = 11) and *BRCA2*-related subgroups (n = 9), respectively. The characters represent cluster memberships of each tumour with *BRCA1 *and *BRCA2 *abnormalities indicated, see bottom of the figure. It can be hypothesised here that component three reflects differences between *BRCA1*- and *BRCA2*-related tumours whereas component two reflects their similarities, see further in Additional data file 3. **(b) **The distribution of genomic instability indices (GII) differed considerably between the identified subgroups in that one of the subgroups displayed low genomic instability whereas the other three displayed high instability levels.

Cluster analysis revealed four distinct subgroups within the set of tumours constituting the whole study group (Figure 2a; see Additional data files 3 and 4). Three of the identified subgroups displayed high levels of genomic instability as measured by the GII, whereas one subgroup was characterised by low instability levels clearly seen in the distribution of GII within this subgroup in comparison with that of the others combined (Wilcoxon rank sum test, *P *< 10^-11^; Figure 2b). One of the GII-high subgroups (n = 11) was enriched with tumours displaying *BRCA1 *abnormalities (6 of 11) defined as an instance of a *BRCA1 *germline mutation or epigenetic silencing of the *BRCA1 *gene (Fisher's exact test, *P *= 0.006). This subgroup will hereafter be referred to as the *BRCA1*-related subgroup. Tumours displaying epigenetic silencing of the *BRCA1 *gene were also highly enriched within this subgroup when sporadic cases were considered exclusively (Fisher's exact test *P *= 0.007). Additionally, two other sporadic tumours within this subgroup displayed loss of BRCA1 protein expression without detectable hypermethylation of the *BRCA1 *gene promoter and both of these tumours were CK5/6 positive. All tumours within this subgroup analysed for loss of heterozygosity at the *BRCA1 *locus displayed allelic imbalance (Fisher's exact test, *P *= 0.003). To validate the relationship with *BRCA1 *abnormalities we obtained previously published CGH array data in which five familial *BRCA1 *breast tumour samples were analysed [27]. The five familial *BRCA1 *tumours were combined with all the samples in our study group to subsequently re-apply the clustering procedure [see additional data file 5]. All of the five familial *BRCA1 *tumours clustered among the tumours that constituted the previously defined subgroup of tumours enriched with *BRCA1 *abnormalities. Combining the two different platforms involves reduction in array resolution for which reasons the five familial *BRCA1 *tumours were not included in subsequent analysis. A total of five blood-derived DNA samples from individuals with sporadic tumours displaying *BRCA1*-like genomic patterns were fully screened for germline mutations in the *BRCA1 *gene and none were found. Another GII-high subgroup (n = 9) was highly enriched of tumours derived from *BRCA2 *germline mutation carriers (8 of 9; Fisher's exact test *P *< 10^-4^; Figures 2a and 2b). We will hereafter refer to this subgroup as the *BRCA2*-related subgroup. The third GII-high subgroup (n = 14) was not related to abnormalities in the *BRCA *genes and will hereafter be referred to as the GII-high-III subgroup (Figures 2a and 2b).

### Genomic alterations characterising the distinct genomic subgroups

The genomic alterations that characterised the *BRCA1*-related subgroup, when compared with the rest of the cohort, were deletions at chromosomes 4p, 4q, 5p/q, Xp, Xq along with copy number gains at 10p and 16q (Fisher's exact test modified, *P *< 0.001) [See Additional data files 6 and 7]. Genomic regions characterising the *BRCA2*-related subgroup were deletions at chromosomes 1p, 3p, 6q, 8p, 11q, 13q, 14q, 16q, 17p and Xp along with copy number gains at 3p, 8q and 17q as compared with the rest of the cohort (Fisher's exact test modified, *P *< 0.001) [see Additional data files 6 and 7]. High-level amplifications at 1q43–q44 and 8q24 were prominent (> 20% of samples) within the *BRCA2*-related subgroup (Fisher's exact test, *P *< 0.05). The one sporadic tumour that clustered among the *BRCA2*-related subgroup displayed gains in copy numbers of the *EMSY *gene located at 11q13.5, which was confirmed by FISH analysis for two different regions of the tumour showing gene/centromere ratios of 1.9 and 3.0, respectively [see Additional data file 8]. Full sequencing of the *BRCA2 *gene was carried out on blood-derived DNA from this individual and no germline mutations were found.

The genomic alterations that characterised the GII-high-III subgroup were mostly small regions of copy number gains [see Additional data files 6 and 7]. High-level amplifications at 11q13.2–q13.3 were prominent (> 20% of samples) within this subgroup (Fisher's exact test, *P *< 0.05). All but two samples (12 of 14; 86%) within this subgroup displayed high- or low-level copy number gains at the 11q13.2–q13.3 genomic region (Fisher's exact test modified, *P *= 0.0003). The high-level amplifications at 11q13.2–q13.3 included two regions at which the level of significance peaks. One of these two regions covered a very small region, about 92 kb, and included a single gene, the *FADD *gene whereas the second region covered about 556 kb and included four genes, that is, *MYEOV*, *CCND1*, *ORAOV1 *and *FGF19*.

The subgroup characterised by tumours with low GIIs was not associated with any specific genomic alterations. Some of these tumours displayed copy number gains at 1q, 8q and 16p and deletions at 8p and 16q. These genomic alterations do not all occur within the same tumour but different combinations of them describe the observed variation in genomic profiles found within this subgroup. It is important to note that we did observe tumours that did not display any of these commonly observed alterations with some having very few copy number alterations. We propose here that these tumours may represent biologically important disease entities of breast cancers.

### Genome architecture patterns

**Figure 3:**
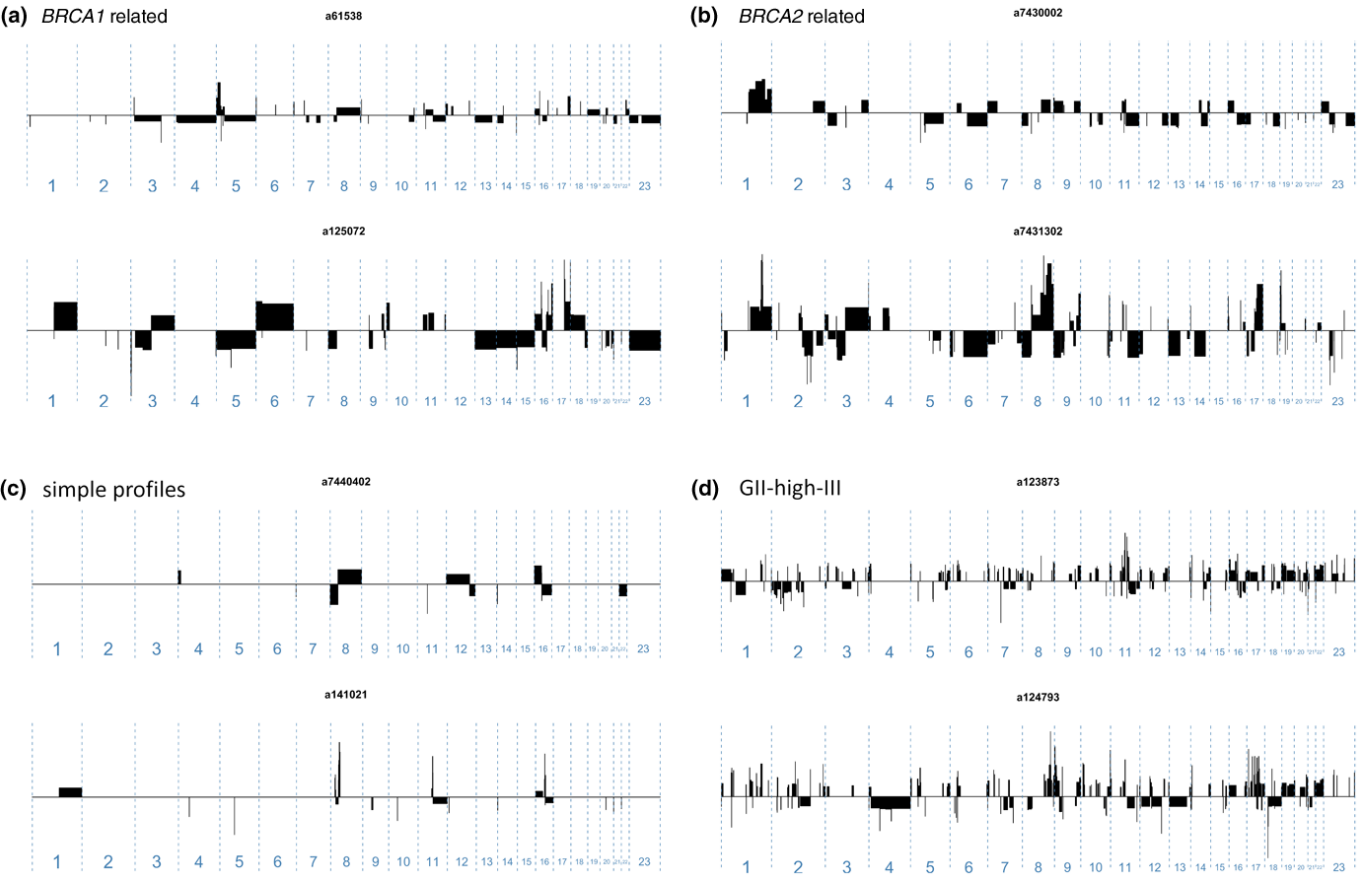
Differences in genome architecture patterns were observed between the identified subgroups. Two tumour genomes are shown for each of the four subgroups. **(a) **The *BRCA1*- and **(b) ***BRCA2*-related subgroups are characterised by relatively long segments of deletions with occasional high-level amplifications. **(c) **The simple-profile subgroup resembled the *BRCA1*- and *BRCA2*-related subgroups but displayed considerably less complex genomes. **(d) **The GII-high-III subgroup was characterised by small and closely spaced segments of genomic alterations throughout their tumour genomes along with occasional high-level amplifications. GII = genomic instability index.

Visual examination of the segmentation profiles revealed clear differences in alteration patterns between each of the identified subgroups, that is, their genome architecture patterns (Figure 3). Tumour genomes within the *BRCA1*- and *BRCA2*-related subgroups were characterised by relatively long stretches of genomic alterations, deletions and copy gains along with occasional high-level amplifications (Figures 3a and 3b). Tumours within the simple-profile subgroup appear similar to the *BRCA1 *and *BRCA2 *subgroups in terms of altered segment lengths but differ in that they display considerably less complex genomes (Figure 3c). The tumours within the GII-high-III subgroup were characterised by numerous closely packed and small copy number alterations throughout their genomes with occasional high-level amplification events (Figure 3d). This is similar to the complex-firestorm patterns described by Hicks and colleagues or the amplification phenotype described by Fridlyand and colleagues whereas the *BRCA1 *and *BRCA2*-related subgroups are more similar to the complex-sawtooth patterns [30,31].

**Figure 4:**
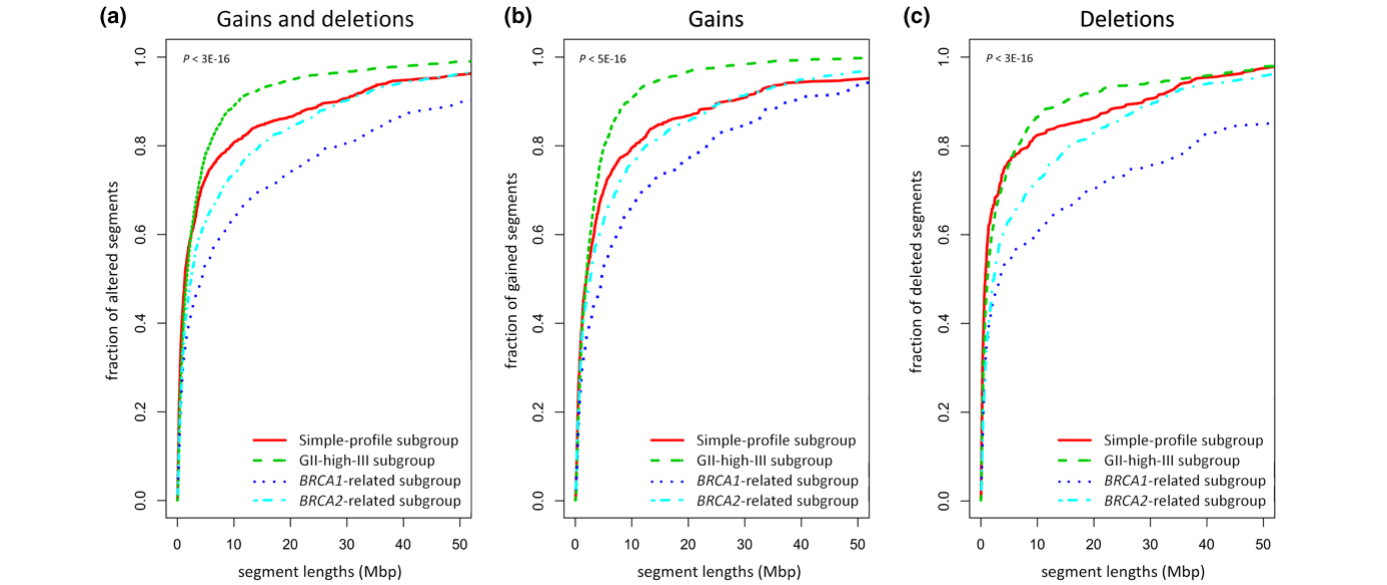
Quantitative analysis of the observed genome architecture patterns. Empirical cumulative distribution curves for segment lengths within each of the four identified subgroups were examined. The distribution of segment lengths for each of the identified subgroups are shown for all segments **(a) **altered in copy number, **(b) **gained in copy number and **(c) **deleted in copy number. The *P*-values in each of these comparisons were highly significant (*P *< 10^-15^) determined through a Kruskal-Wallis hypothesis test.

The observed phenotypic features of the tumour genomes were quantitatively analysed by examining the segment lengths within each subgroup. This analysis demonstrates that the distribution of segment lengths within the GII-high-III subgroup is shifted towards smaller segments, whereas the tumours within the *BRCA1*-related subgroup display a shift towards longer segments (Figure 4a). Examining the segment lengths of deletions and gains separately shows that the *BRCA1*- and *BRCA2*-related subgroups are characterised by large deletions whereas the GII-high-III subgroup is characterised by small copy number gains rather than deletions (Figures 4b and 4c). Pair-wise comparisons for the distributions in deleted segment lengths between subgroups demonstrates that each of the *BRCA1*- and *BRCA2*-related subgroups are significantly different from the simple- and/or GII-high-III subgroups (Wilcoxon rank sum test, *P *< 10^-11^).

### Tumour phenotypes and their relation with genomic profiles

The relation between the identified genomic subgroups and tumour phenotypes was examined using a selected panel of biomarkers analysed on TMAs. Tumour phenotypes were established as described in Cheang and colleagues by expression analysis of five biomarkers, ER, PR, HER-2, EGFR and CK5/6 on TMA sections [25]. Additionally, we examined the expression of CK8 and CK18 on these TMAs.

**Figure 5:**
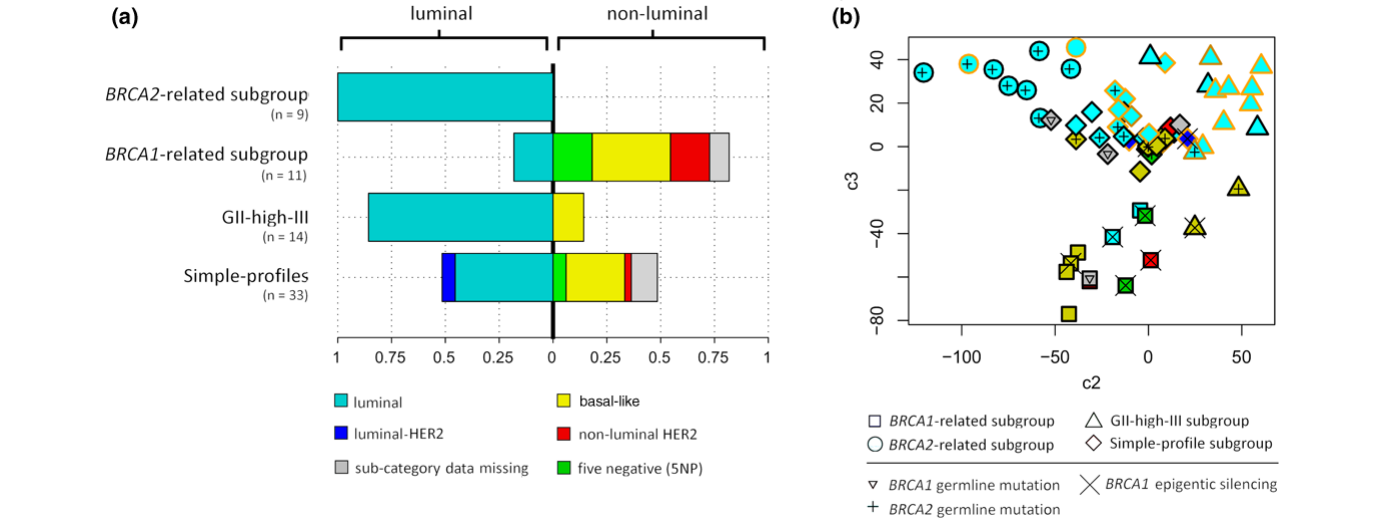
Tumour phenotypes in relation to the identified genomic subgroups. **(a) **Expression of oestrogen-receptor (ER), progesterone receptor (PR) and human epidermal growth factor receptor (HER)-2 was available in all cases enabling classification of all tumours as luminal or non-luminal. The tumours were then further subdivided using the five biomarker scheme based on expression analysis of ER, PR, HER-2, epidermal growth factor receptor (EGFR) and cytokeratin (CK) 5/6. The left part of each rectangle represents the proportion of luminal phenotypes within each of the four subgroups, as indicated, whereas the right part represents the proportion of non-luminal tumours. Sub-categories of these phenotypes are represented there within as proportions of either luminal or non-luminal phenotypes, see bottom of the figure. **(b) **Projection of all tumours through principal component analysis (PCA) is shown with cluster outcomes, *BRCA *status and the assigned tumour phenotypes indicated. Colours indicate tumour phenotypes matching those given at the bottom left panel with the addition of high PR expression immunohistochemistry scores 2+ and ≥ 3+ indicated by dark and light orange character outlines, respectively. Cluster memberships and *BRCA *status are indicated as shown at the bottom of the figure. In terms of tumour phenotypes, it can be hypothesised that component three reflects differences in luminal *vs*. non-luminal phenotypes whereas component two separates two populations of luminal tumours, which relate to differential PR expression.

Clear trends for particular phenotypic properties were observed for the three genomic instability groups (GII-high; Figures 5a and 5b) [see Additional data files 3 and 9]. The *BRCA1*-related subgroup was enriched for tumours displaying non-luminal phenotypes (9 of 11, 82%; Fisher's exact test, *P *= 0.0049) and grade 3+ (8 of 11, 73%). Of the nine non-luminal tumours within this subgroup a total of eight were fully interpretable for all the five biomarkers and were thus further subdivided into basal-like (4 of 8, 50%), non-luminal HER-2 (2 of 8, 25%) and tumours negative for all five biomarkers, 5NP, (2 of 8, 25%; Figure 5a). The two luminal tumours within this subgroup displayed high ER expression (3+) and negativity for HER-2 amplification. The *BRCA2*-related subgroup was entirely composed of luminal tumours (9 of 9, 100%). All but one of the tumours within this subgroup displayed high expression of ER (≥ 2+; 8 of 9, 89%) and almost all were of grade 3+ (4 of 5, 80%). Four of the seven tumours displaying hyper-intense ER staining (> 3+) were found within this subgroup (4 of 7, 57%; Fisher's exact test *P *= 0.0049). All of the nine tumours within this subgroup were HER-2 negative (9 of 9, 100%; Figure 5a). The GII-high-III subgroup was almost entirely composed of luminal tumours (12 of 14, 86%). This group of tumours displayed an unusually high frequency of high PR expression with IHC score 3+ (7 of 14, 50%; Fisher's exact test *P *= 0.034) and with IHC score ≥ 2+ (9 of 14, 64%; Fisher's exact test *P *= 0.0072; Figure 5b). Supporting this observation is the finding that two of the three tumours within the entire study group displaying hyper-intense staining of the PR gene (> 3+) were found within the GII-high-III subgroup. It can be hypothesised here that the third component shown in Figure 5b reflects differences in luminal *vs*. non-luminal phenotypes whereas the second component splits up two populations of luminal tumours that are different in terms of PR expression (Figure 5b) [see Additional data file 3].

The simple-profiles subgroup was found to represent a heterogeneous group of tumours in terms of their phenotypes. An important observation is that the luminal tumours within this subgroup displayed a trend towards lower tumour grade as compared with the luminal tumours within the more complex GII-high subgroups (Chi-squared test for trend, *P *= 0.032). However, non-luminal tumours within the simple-profile subgroup displayed a trend towards high tumour grade as compared with the luminal tumours within the same subgroup (Chi-squared test for trend, *P *= 0.0015).

### Tumours displaying low genomic instability indices

**Figure 6:**
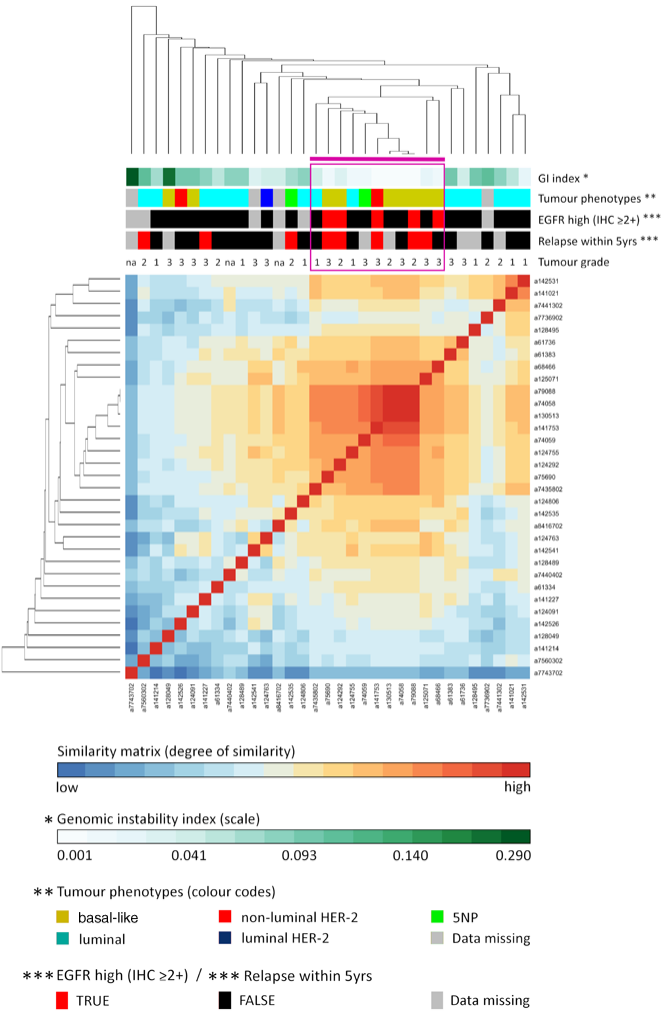
Hierarchical cluster analysis of genomic profiles within the simple-profiles subgroup. This analysis revealed a cluster of tumours (n = 11), purple bar and rectangle, characterised by very low genomic instability indices. This cluster was highly enriched with tumours of basal-like phenotypes with high expression of epidermal growth factor receptor (EGFR) being a prominent feature. These tumours generally displayed high tumour grade and an aggressive phenotype in terms of time to relapse.

The observed heterogeneity within the simple-profile subgroup was further examined by hierarchical cluster analysis on the genomic profiles found within this subgroup. This analysis revealed a cluster of tumours (n = 11) characterised by very low genomic instability indices (mean of 0.029 ± 0.026) as compared with the other simple-profile tumours (Wilcoxon rank sum test, *P *< 10^-5^) and the rest of the cohort (Wilcoxon rank sum test, *P *< 10^-6^). This cluster of tumours displaying mostly 'flat' genomes included high frequency of non-luminal phenotypes (9 of 11, 82%) of which most were basal-like (7 of 9, 78%) and grade 3+ (Figure 6). Although they are characterised by silent or flat genomes, these tumours occasionally show small spikes of alterations, including focal high-level amplifications and very small deletions. We observed that this cluster of silent-tumours (n = 11), referred to hereafter using the 'silent' prefix, more frequently displayed high expression of EGFR (≥ 2+) gene products as compared with the rest of the cohort (Fisher's exact test, *P *= 0.0069). Two of these five tumours with high EGFR expression displayed high-level amplification of the *EGFR *gene, which was not found in any other tumour within the study group [see Additional data file 10]. Importantly, the non-luminal tumours with silent genomes displayed an aggressive phenotype in terms of disease outcome (Figure 6). By contrast, the luminal tumours within the simple-profile subgroup displayed low tumour grade and few of these patients experienced relapse within 5 years suggesting non-aggressive disease (Figure 6).

Tumours derived from *BRCA1 *germline mutation carriers were not found within the cluster of 11 tumours displaying silent genomes. However, two familial *BRCA2 *tumours were found within this cluster, neither of which displayed deletion or allelic imbalance at the *BRCA2 *locus and both had an extremely low GII, that is less than 0.034, and were of basal-like phenotype.

## Discussion

The results presented here portray detailed views of genomic alterations in breast cancers and their relation with *BRCA *abnormalities and tumour phenotypes. The purpose of this study was to examine the potential involvement of the *BRCA1 *and *BRCA2 *genes in sporadic breast tumour development.

### A subset of sporadic tumours develop either BRCA1- or BRCA2-like patterns of genomic alterations

Classification of genomic profiles through cluster analysis revealed four distinct subgroups of which two displayed high prevalence of tumours having either *BRCA1*- or *BRCA2 *abnormalities. These two subgroups, referred to as the *BRCA1*- and *BRCA2*-related subgroups respectively, displayed distinct patterns of genomic alterations and high instability indices. Importantly, our results show that sporadic tumours with epigenetic silencing of the *BRCA1 *gene develop similar patterns of genomic alterations as tumours derived from *BRCA1 *germline mutation carriers. This suggests that inactivation of the *BRCA1 *gene through epigenetic silencing is an important event in sporadic breast tumour development. We found two tumours within this genomic subgroup displaying loss of BRCA1 expression without promoter methylation of the *BRCA1 *gene and both of these tumours expressed the basal marker CK5/6. Given the observations described above, it is reasonable to speculate that sporadic tumours displaying *BRCA1*-like genomic alterations are promoted by defects linked with the *BRCA1 *gene function in genomic maintenance.

Interestingly, one sporadic tumour classified among the *BRCA2*-related subgroup, which was otherwise exclusively comprised of tumours derived from *BRCA2 *germline mutation carriers. This tumour displayed a deletion at the *BRCA2 *gene locus and gains in *EMSY *gene copy numbers. EMSY gene products are known to interact with and negatively regulate *BRCA2 *proteins and this may link the *BRCA2 *gene with sporadic breast tumour development. Obviously, further research is needed to examine the relation between *EMSY *amplification and *BRCA2*-like patterns of genomic alterations.

### BRCA-like genomic instability

Although *BRCA1*- and *BRCA2*-related tumours develop through alterations affecting different regions in their genomes they showed similarities in their genomic architecture patterns with large segments of deletions being prominent. This suggests a similar mechanism by which these tumours acquire instability and we propose here that this might relate to the involvement of the *BRCA1 *and *BRCA2 *genes in error-free DNA repair of double-strand breaks through HR (homologous recombination). Inactivation of either *BRCA1 *or *BRCA2 *is generally thought to lead to the repair of double-strand breaks by error-prone mechanisms via non-homologous end joining [32,33]. DNA repair of double-strand breaks by non-homologous end joining can lead to errors leading to gains or losses of large segments of genomic material. This mechanism could underlie the characteristic type of genomic instability observed within the *BRCA1*- and *BRCA2*-related subgroups. Alternative but not mutually exclusive interpretations relate to the proposed roles of the *BRCA *genes in telomere maintenance and centrosome division [4,34,35]. By contrast, the third instability subgroup, GII-high-III, was found to display increased propensity to acquire small copy number gains which might relate to the previously proposed 'amplifier phenotypes' and possibly 'complex-firestorm patterns' in breast tumours [27,31]. The 7 kbp high-resolution array CGH analysis used in this study is crucial for distinguishing between tumour genomes characterised by small copy gains from those displaying large-scale instability patterns. This provided increased clarity in classification of breast tumours by their genomic profiles.

### Genomic alterations characterizing BRCA1- and BRCA2-related tumour development

The genomic regions on chromosomes 4, 5 and 10 reported here to characterise the *BRCA1*-related subgroup overlap with those previously reported to distinguish tumours derived from *BRCA1 *germline mutation carriers [36,37]. Because familial *BRCA1 *tumours resemble basal-like tumours in terms of their phenotype it is of interest to note here that the genomic alterations that characterise the *BRCA1*-related subgroup overlap with those associated with basal-like tumours [19].

The genomic alterations that were found to characterise *BRCA2*-related tumour development overlap with those previously described in relation with familial *BRCA2 *tumours [36,38]. In addition, we found high-level amplifications at 1q43–q44 and deletions at chromosome 14q, which have not been described before in relation with familial *BRCA2 *tumour development. The detailed information on the locations of genomic alterations provided by the high-resolution CGH arrays used here allowed us to more clearly delineate the distinct genetic pathways undertaken by breast tumours displaying either *BRCA1 *or *BRCA2 *abnormalities.

Further research will be needed to examine the potential of the data presented here to predict *BRCA1 *or *BRCA2 *abnormalities in an independent population of breast tumours or cell lines. The importance of establishing a simple and effective classification scheme to identify such tumours lies in the potential benefit of targeted therapy (PARP inhibitors, platinum drugs) for a much larger group of patients than the relatively few *BRCA1 *and *BRCA2 *germline mutation carriers.

### Tumour phenotypes in BRCA-related tumour development

Tumours derived from *BRCA1 *germline mutation carriers have previously been shown to predominantly display basal-like phenotypes [17,39]. In line with this we observed that tumours within the *BRCA1*-related subgroup primarily display non-luminal phenotypes of which basal-like phenotypes were the most prominent. However, we did identify a cluster of tumours characterised by low genomic instability indices and non-luminal phenotypes. These tumours were mostly of basal-like phenotypes and displayed an aggressive phenotype in terms of disease outcome. This observation demonstrates that a subset of non-luminal breast tumours do not develop towards large-scale genomic alterations supporting the hypothesis that these tumours represent biologically important disease entities [20]. Tumours derived from *BRCA2 *germline mutation carriers have previously been shown to primarily display luminal phenotypes and rarely overexpress *HER-2 *gene products and these findings were confirmed here [40]. Interestingly, we observed two familial-*BRCA2 *tumours without deletion or allelic imbalance at the *BRCA2 *locus and these tumours did not display large-scale genomic instability. This raises the possibility that the natural history of some familial *BRCA2 *tumours does not involve loss of the wild-type *BRCA2 *allele or at least only partial loss as has been suggested before [41]. In this relation, it has been shown that cells heterozygous for a *BRCA2 *mutation are associated with a phenotype [42,43]. Taken together, this suggests that a small subset of *BRCA2 *tumours could be promoted by haploinsufficiency for the *BRCA2 *gene.

The combined analysis of genomic alterations and tumour phenotypes, presented here, show that *BRCA1*- and *BRCA2*-related tumours develop largely through different genetic pathways in terms of the regions altered, while also displaying distinct phenotypes. In light of the common roles for *BRCA1 *and *BRCA2 *in genomic maintenance, this suggests that the observed phenotypic differences impose selective advantages for genomic alterations at distinct regions in the context of instability generated by *BRCA*-deficiency. This is in agreement with the results described by Melchor and colleagues showing the importance of ER status in familial and sporadic breast tumours [44]. However, we found two genomically distinct populations of luminal tumours that clearly differed in terms of PR expression. This finding is novel and demonstrates the importance of this factor in breast tumour development.

## Conclusions

We have demonstrated using high-resolution genomic profiling coupled with analysis of tumour phenotypes that the development of a subset of sporadic breast tumours is similar to that of tumours derived from *BRCA1*- or *BRCA2 *germline mutation carriers. Tumours that develop *BRCA1*-like patterns of genomic alterations predominantly displayed high-grade, non-luminal phenotypes and high genomic instability. However, we also found a subset of high grade non-luminal tumours, mostly basal-like, that displayed very silent genomes characerised by low genomic instability indices supporting the notion of a novel subgroup of ER-negative breast tumours [20,45].

Tumours within the *BRCA1*- and *BRCA2*-related genomic subgroups were found to acquire genomic alterations affecting distinct regions of their genomes while also displaying distinct tumour phenotypes. Given the common roles of the *BRCA *gene products in genomic maintenance, this suggests that phenotypic differences between *BRCA1*- and *BRCA2*-associated tumours impose selective advantages for distinct genomic alterations in the context of instability generated by *BRCA*-deficiency. Despite these differences, the *BRCA1 *and *BRCA2 *genomic subgroups displayed clear similarities in their genome architecture patterns where large deletions were prominent suggesting a similar mechanism by which genomic instability is brought about, possibly relating to defects in DNA repair through HR. This genomic feature was observed in both familial and sporadic tumours displaying a *BRCA1*- or *BRCA2*-like spectrum of genomic alterations. In this respect, it has been shown that cells with defective DNA repair by HR, including *BRCA*-deficient cells, are sensitive to agents that lead to DNA double-strand breaks such as PARP inhibitors and platinum agents [9]. The importance of the results presented here involve the potential benefits of targeted therapy through the use of agents that lead to double-strand breaks for a larger group of patients than the relatively few *BRCA *germline mutation carriers [46,47].

## Abbreviations

CBS: circular binary segmentation; CGH: comparative genomic hybridisation; CK: cytokeratin; EGFR: epidermal growth factor receptor; ER: oestrogen receptor; FISH: fluorescence *in situ *hybridiaation; GII: genomic instability index; HER: human epidermal growth factor receptor; IHC: immunohistochemisty; MSP: methylation-specific PCR; PCA: principal component analysis; PR: progesterone receptor; TMA: tissue microarrays.

## Competing interests

The authors declare that they have no competing interests.

## Authors' contributions

OAS contributed to the study design and performed the MSP and CGH analysis along with statistical analysis, data mining and writing of the manuscript. KO constructed the tissue microarrays and performed the IHC analysis. JGJ and OAS scored the IHC results. JGJ analysed tumour grade and OTJ contributed to clinical data analysis. MS performed the FISH analysis. SV contributed to the CGH analysis. JE conceived of the study, was in charge of its design and coordination and writing of the manuscript. All authors read and approved of the manuscript.

## Supplementary Material

Additional file 1 An Excel file containing a table that lists antibodies used in the study for immunohistochemistry analysis on tissue microarray sections. Click here for file

Additional file 2 A TIF file containing a figure that lists the proportion of the variance in the genomic data explained by each of the components derived from the principal component analysis (PCA). Click here for file

Additional file 3 A TIF file containing a figure that lists the projection of all tumours on components 1, 2 and 3 through principal component analysis (PCA) is shown with cluster outcomes, *BRCA *status and tumour phenotypes indicated. Click here for file

Additional file 4 An Excel file containing a sample annotation table describing the cluster outcomes in terms of *BRCA *status, AI at the *BRCA1 *and *BRCA2 *loci, genomic instability index along with tumour phenotypes. Click here for file

Additional file 5 A TIF file containing a figure that shows genomic profiles derived from an independent set of five familial *BRCA1 *tumours were combined with the study group. All of these five familial *BRCA1 *tumours, indicated in grey colour, clustered among the tumours that constituted the previously defined *BRCA1*-related subgroup. The character codes represent cluster memberships with the five familial *BRCA1 *tumours included whereas the colour codes represent previously defined cluster memberships as shown on Figure 2a in the manuscript. Tumours derived from *BRCA1 *and *BRCA2 *germline mutation carriers are indicated, see bottom of the figure. Click here for file

Additional file 6 An Excel file containing a table that lists genomic alterations characterising the distinct genetic pathways that were identified through cluster analysis of genomic profiles. Click here for file

Additional file 7 A TIF file containing a figure that lists genomic alterations characterising each of the identified genetic pathways visualised using a frequency plot. The proportion of tumours showing gains (positive) and deletions (negative) are shown for each of the genomic regions examined. Additionally, the level of statistical significance is shown as determined through the modified Fisher's exact test comparing each genomic subgroup with the rest of the cohort. Click here for file

Additional file 8 A TIF file containing a figure that shows (upper panel) gains in copy numbers of the EMSY gene (11q13.5) observed in one sporadic tumour displaying *BRCA2*-like patterns of genomic alterations. (lower panel) fluorescence *in situ *hybridisation (FISH) analysis was performed for the *EMSY *gene region (RED) and centromere 11 (GREEN) verifying amplification of the *EMSY *gene in this tumour. Click here for file

Additional file 9 A TIF file containing a figure that shows hierarchical cluster analysis of the biomarkers examined by immunohistochemistry on tissue microarray sections. Tumour phenotypes were established through analysis of these markers using the five biomarker scheme. The assigned tumour phenotypes are indicated on top of each heat map. See colour codes at the bottom of the figure. Click here for file

Additional file 10 A TIF file containing a figure that shows high expression of epidermal growth factor receptor (EGFR) gene products (only membrane staining was scored) in four of the nine non-luminal tumours displaying 'silent' genomes. High-level amplifications of the *EGFR *gene were found in two of these tumours and gain of the entire chromosome 7, where the *EGFR *gene resides, was found in one of them. High-level amplifications of the *EGFR *gene were not found anywhere else within the entire study group. **(a) **High expression of EGFR gene products (≥ 2+) in four example tumours, all of which displaying 'silent' genomes. The immunohistochemistry scores are indicated in each case. **(b) **High-level amplifications of the *EGFR *gene were found in two tumours displaying 'silent' genomes viewed here in 37.5 kb resolution (5×). **(c) **Chromosome 7 viewed in 7 kbp resolution (1×) of the same example genome with high-level amplification of the *EGFR *gene indicated. Click here for file
