# Supplementary figures and images for: Genomic profiling of breast tumours in relation to BRCA abnormalities and phenotypes
Source: Breast Cancer Res. 2009 Jul 9;11(4):R47. doi: 10.1186/bcr2334 (PMC2750106; doi:10.1186/bcr2334)

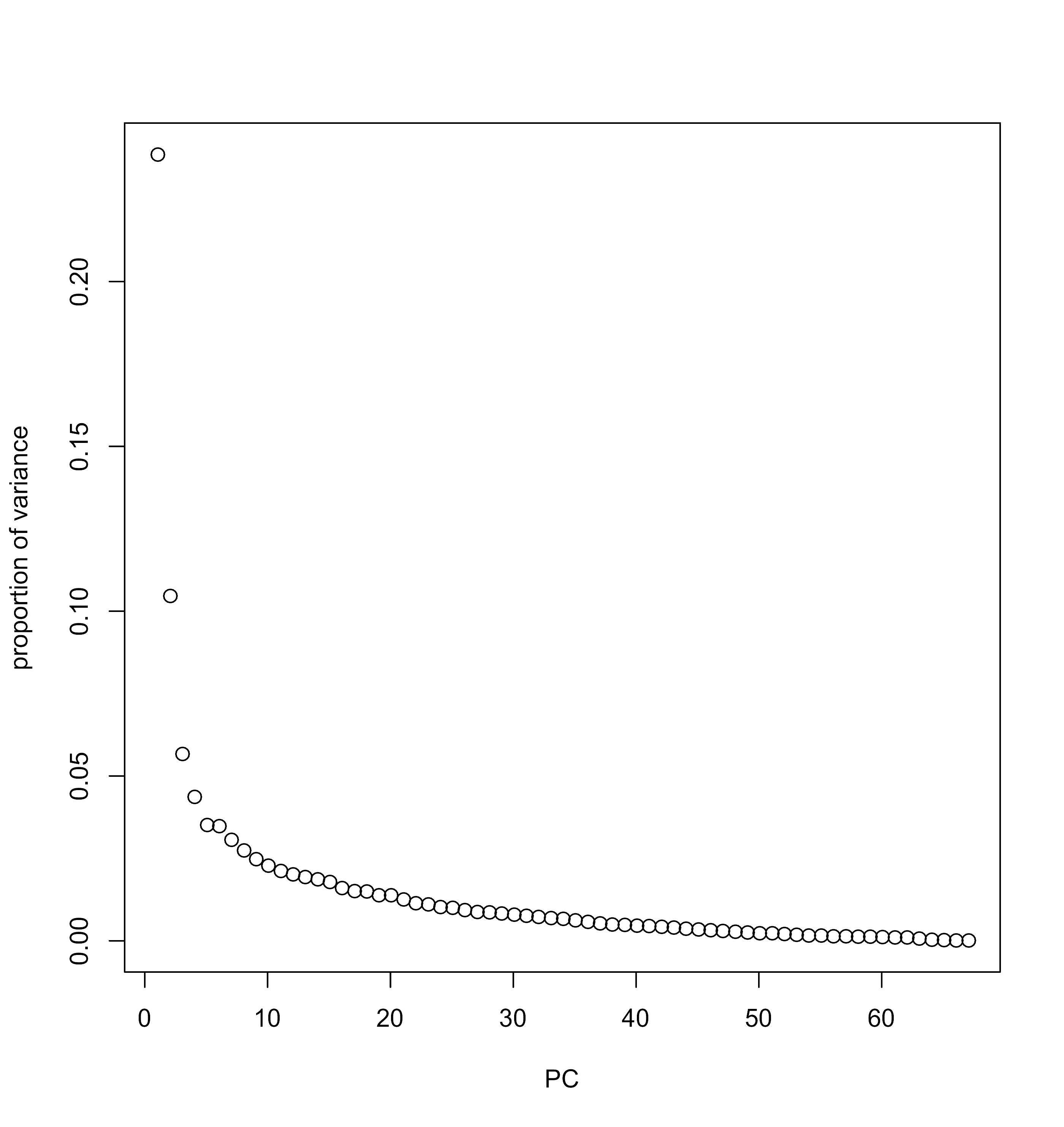

Supplement: Additional file 2 — A TIF file containing a figure that lists the proportion of the variance in the genomic data explained by each of the components derived from the principal component analysis (PCA). [file bcr2334-S2.tiff]

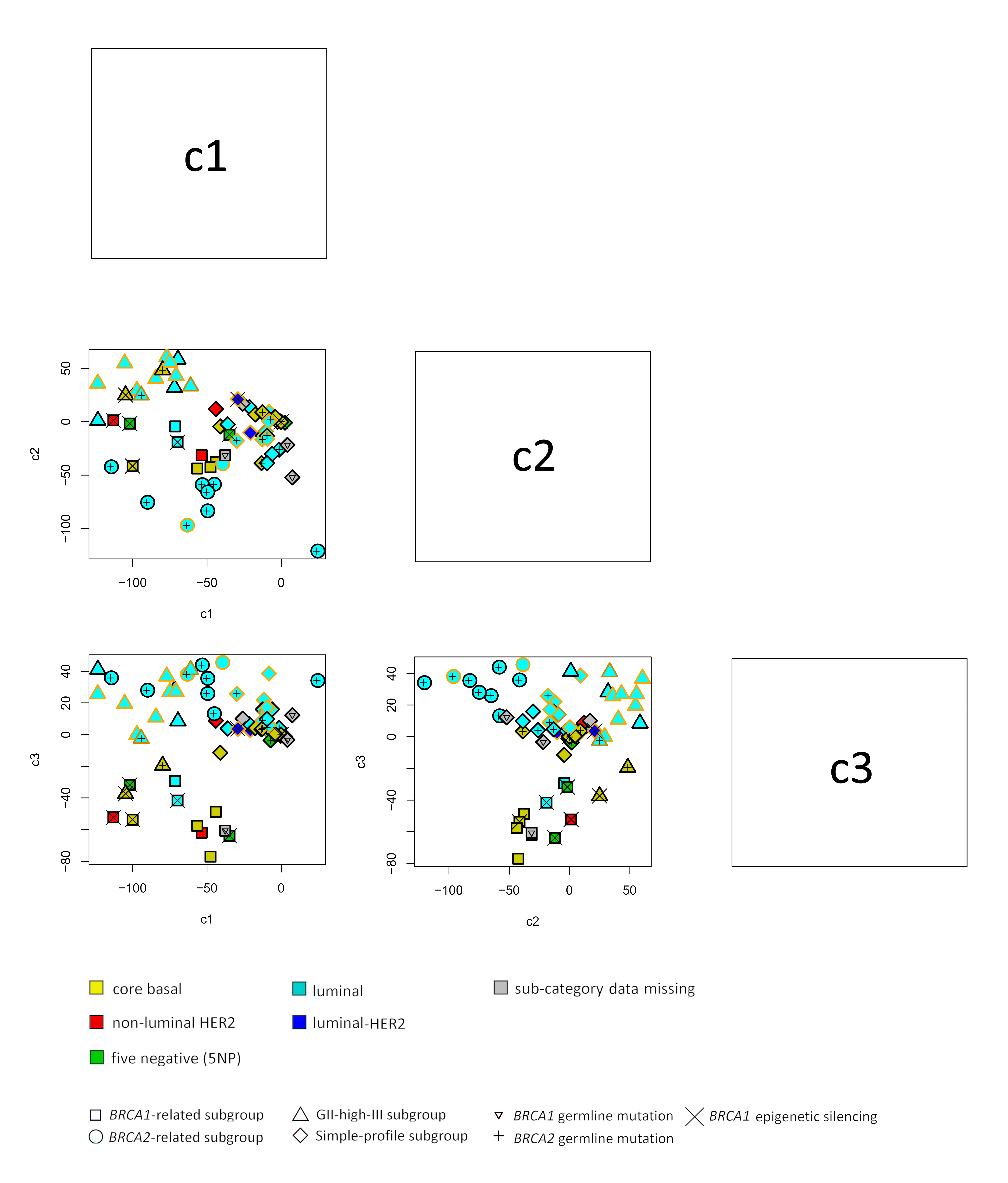

Supplement: Additional file 3 — A TIF file containing a figure that lists the projection of all tumours on components 1, 2 and 3 through principal component analysis (PCA) is shown with cluster outcomes, BRCA status and tumour phenotypes indicated. [file bcr2334-S3.tiff]

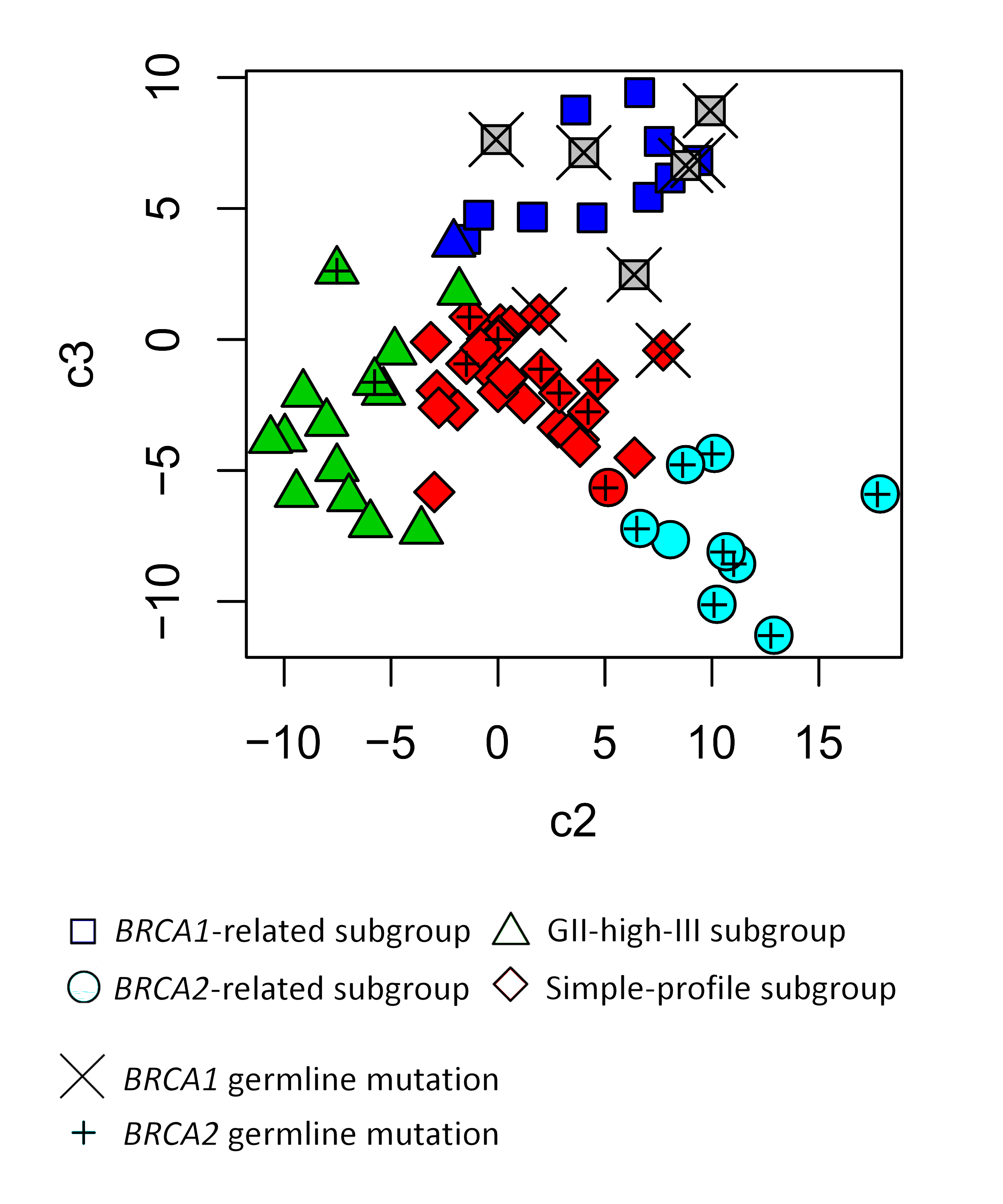

Supplement: Additional file 5 — A TIF file containing a figure that shows genomic profiles derived from an independent set of five familial BRCA1 tumours were combined with the study group. All of these five familial BRCA1 tumours, indicated in grey colour, clustered among the tumours that constituted the previously defined BRCA1-related subgroup. The character codes represent cluster memberships with the five familial BRCA1 tumours included whereas the colour codes represent previously defined cluster memberships as shown on Figure 2a in the manuscript. Tumours derived from BRCA1 and BRCA2 germline mutation carriers are indicated, see bottom of the figure. [file bcr2334-S5.tiff]

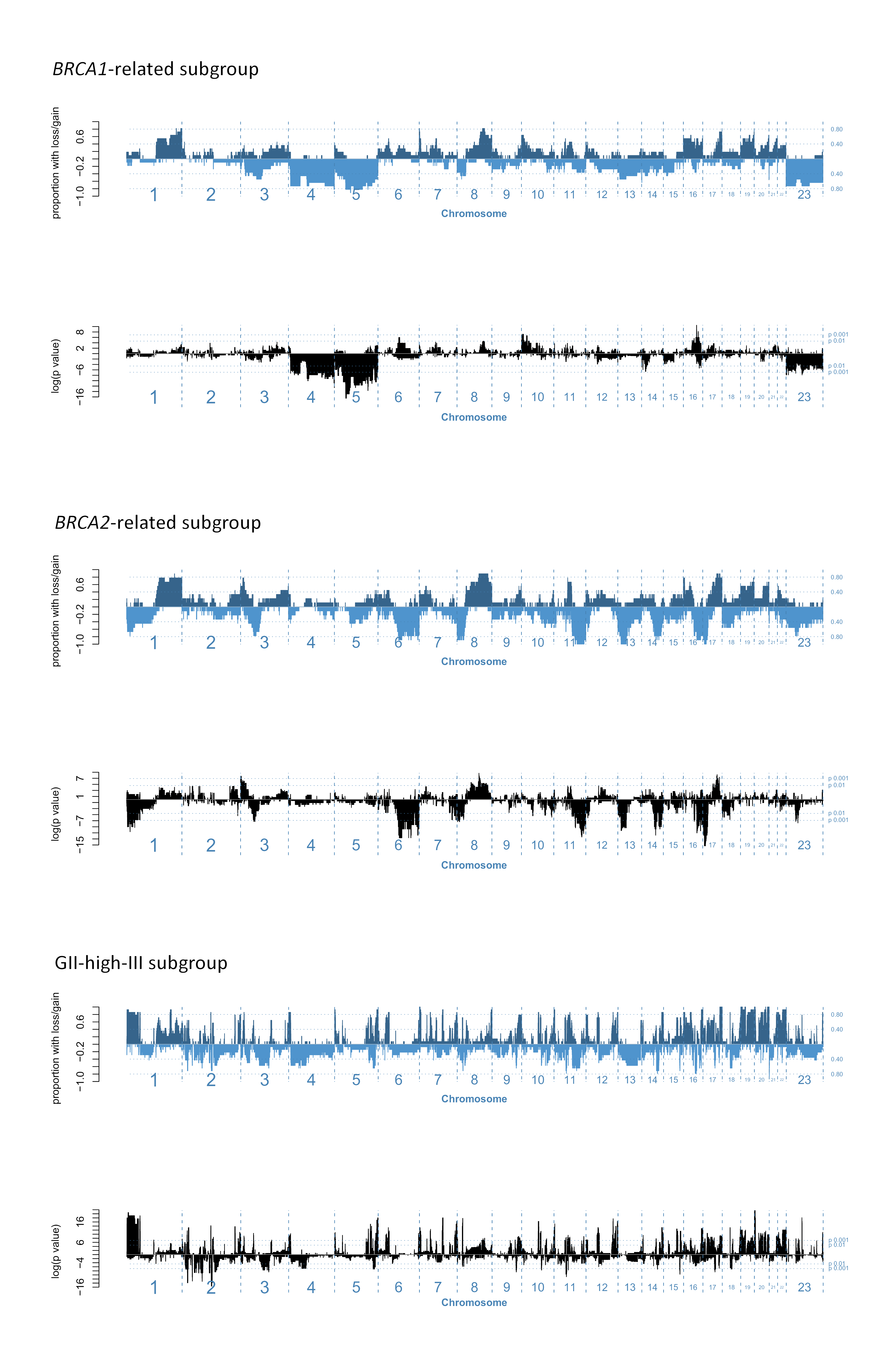

Supplement: Additional file 7 — A TIF file containing a figure that lists genomic alterations characterising each of the identified genetic pathways visualised using a frequency plot. The proportion of tumours showing gains (positive) and deletions (negative) are shown for each of the genomic regions examined. Additionally, the level of statistical significance is shown as determined through the modified Fisher's exact test comparing each genomic subgroup with the rest of the cohort. [file bcr2334-S7.tiff]

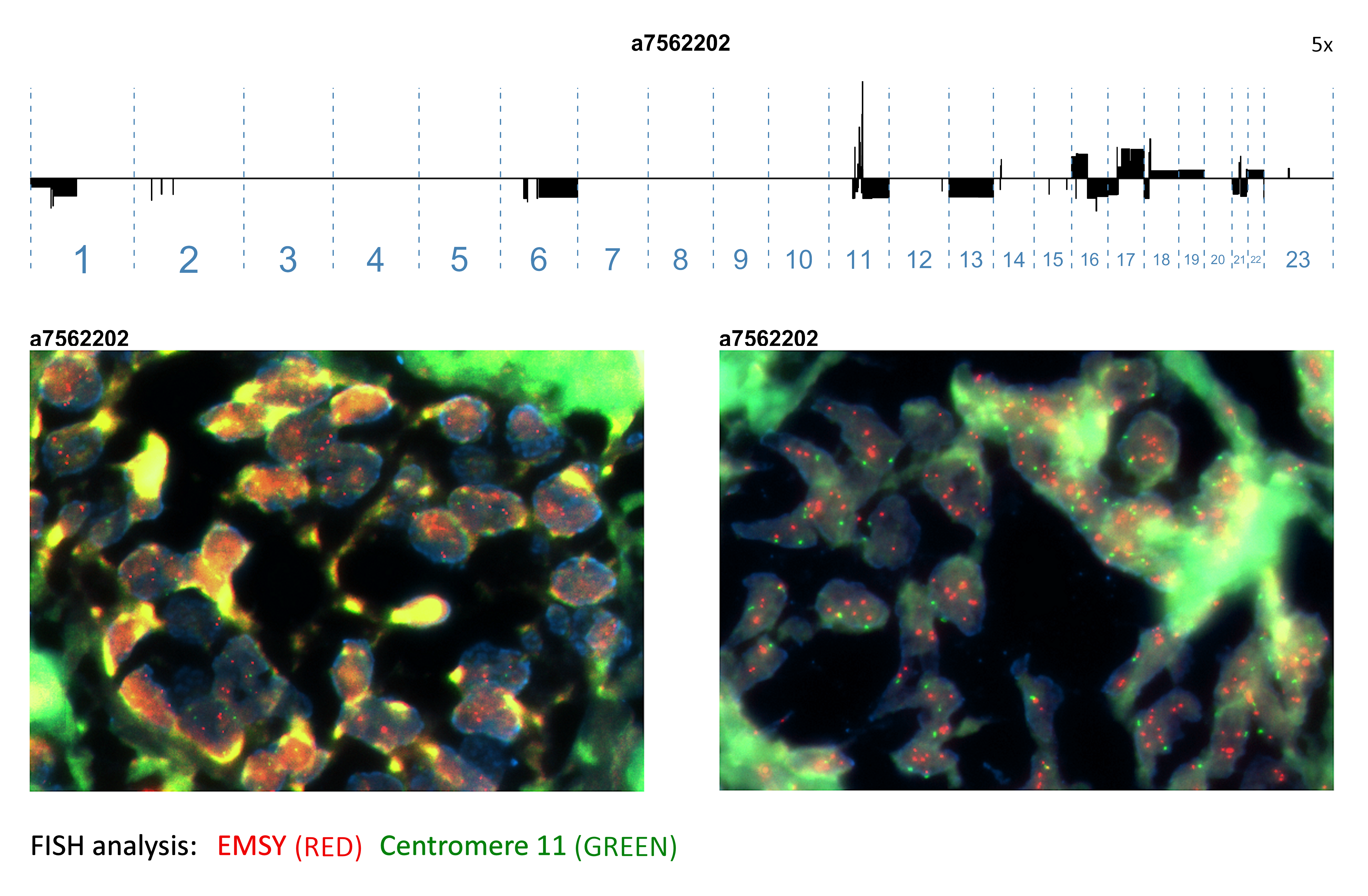

Supplement: Additional file 8 — A TIF file containing a figure that shows (upper panel) gains in copy numbers of the EMSY gene (11q13.5) observed in one sporadic tumour displaying BRCA2-like patterns of genomic alterations. (lower panel) fluorescence in situ hybridisation (FISH) analysis was performed for the EMSY gene region (RED) and centromere 11 (GREEN) verifying amplification of the EMSY gene in this tumour. [file bcr2334-S8.tiff]

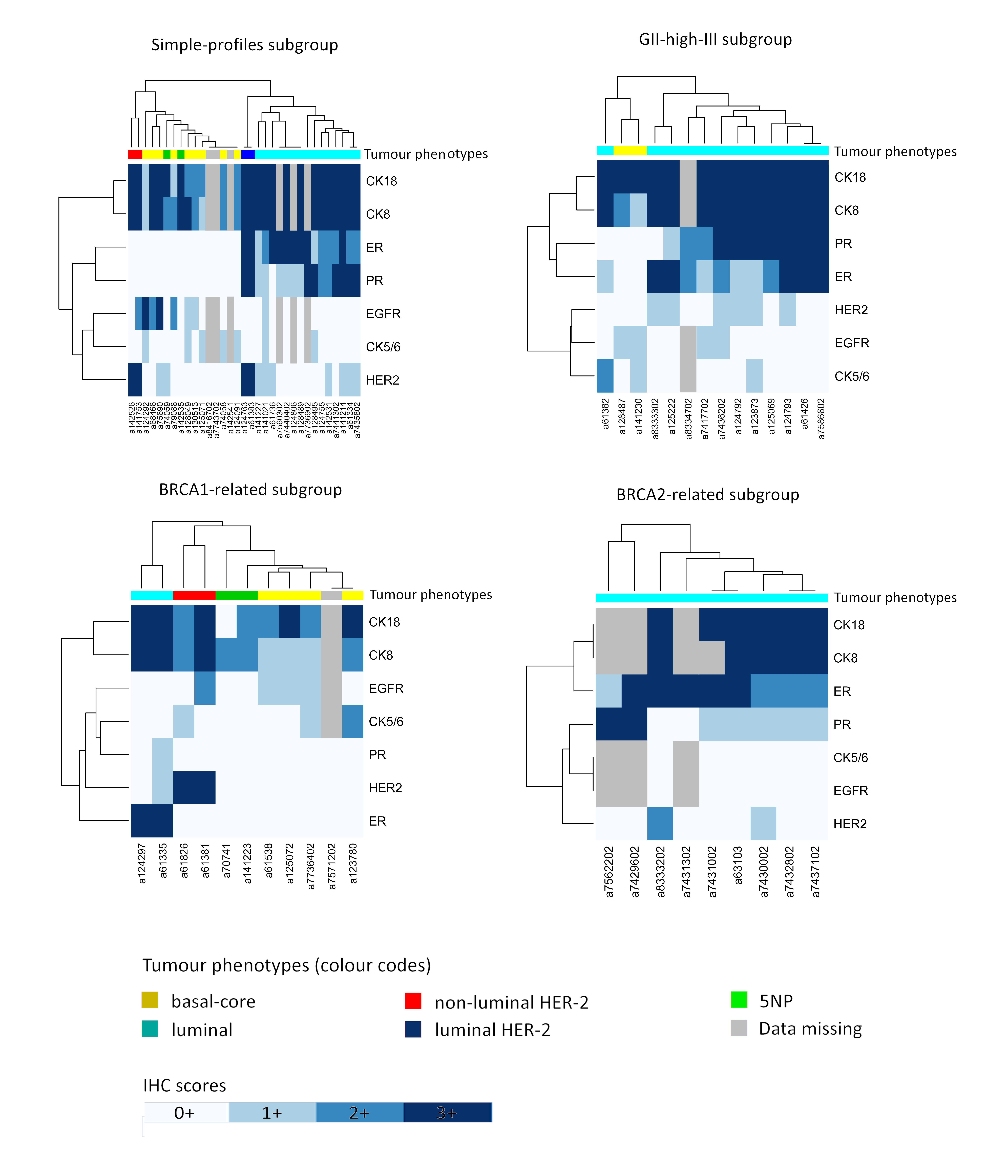

Supplement: Additional file 9 — A TIF file containing a figure that shows hierarchical cluster analysis of the biomarkers examined by immunohistochemistry on tissue microarray sections. Tumour phenotypes were established through analysis of these markers using the five biomarker scheme. The assigned tumour phenotypes are indicated on top of each heat map. See colour codes at the bottom of the figure. [file bcr2334-S9.tiff]

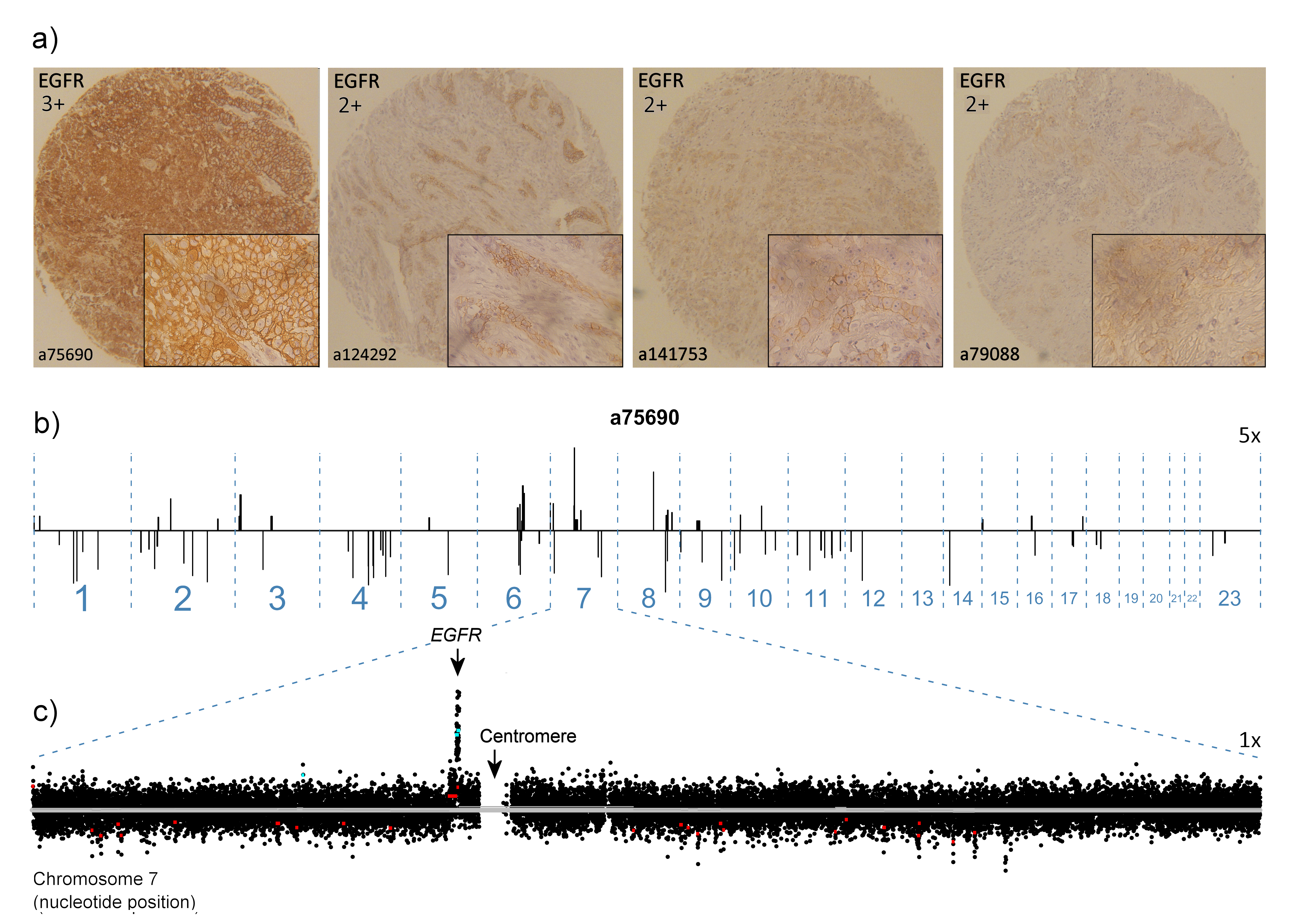

Supplement: Additional file 10 — A TIF file containing a figure that shows high expression of epidermal growth factor receptor (EGFR) gene products (only membrane staining was scored) in four of the nine non-luminal tumours displaying 'silent' genomes. High-level amplifications of the EGFR gene were found in two of these tumours and gain of the entire chromosome 7, where the EGFR gene resides, was found in one of them. High-level amplifications of the EGFR gene were not found anywhere else within the entire study group. (a) High expression of EGFR gene products (≥ 2+) in four example tumours, all of which displaying 'silent' genomes. The immunohistochemistry scores are indicated in each case. (b) High-level amplifications of the EGFR gene were found in two tumours displaying 'silent' genomes viewed here in 37.5 kb resolution (5×). (c) Chromosome 7 viewed in 7 kbp resolution (1×) of the same example genome with high-level amplification of the EGFR gene indicated. [file bcr2334-S10.tiff]
